# The Lasting Influences of Early Food-Related Variety Experience: A Longitudinal Study of Vegetable Acceptance from 5 Months to 6 Years in Two Populations

**DOI:** 10.1371/journal.pone.0151356

**Published:** 2016-03-11

**Authors:** Andrea Maier-Nöth, Benoist Schaal, Peter Leathwood, Sylvie Issanchou

**Affiliations:** 1 Nestlé Research Center, PO Box 44, CH-1000, Lausanne, 26, Switzerland; 2 CNRS, UMR6265 Centre des Sciences du Goût et de l'Alimentation, F-21000, Dijon, France; 3 INRA, UMR1324 Centre des Sciences du Goût et de l'Alimentation, F-21000, Dijon, France; 4 Univ. Bourgogne Franche-Comté, UMR Centre des Sciences du Goût et de l'Alimentation, F-21000, Dijon, France; Chiba University Center for Forensic Mental Health, JAPAN

## Abstract

Children’s vegetable consumption falls below current recommendations, highlighting the need to identify strategies that can successfully promote better acceptance of vegetables. Recently, experimental studies have reported promising interventions that increase acceptance of vegetables. The first, offering infants a high variety of vegetables at weaning, increased acceptance of new foods, including vegetables. The second, offering an initially disliked vegetable at 8 subsequent meals markedly increased acceptance for that vegetable. So far, these effects have been shown to persist for at least several weeks. We now present follow-up data at 15 months, 3 and 6 years obtained through questionnaire (15 mo, 3y) and experimental (6y) approaches. At 15 months, participants who had been breast-fed were reported as eating and liking more vegetables than those who had been formula-fed. The initially disliked vegetable that became accepted after repeated exposure was still liked and eaten by 79% of the children. At 3 years, the initially disliked vegetable was still liked and eaten by 73% of the children. At 6 years, observations in an experimental setting showed that children who had been breast-fed and children who had experienced high vegetable variety at the start of weaning ate more of new vegetables and liked them more. They were also more willing to taste vegetables than formula-fed children or the no or low variety groups. The initially disliked vegetable was still liked by 57% of children. This follow-up study suggests that experience with chemosensory variety in the context of breastfeeding or at the onset of complementary feeding can influence chemosensory preferences for vegetables into childhood.

## Introduction

There is considerable evidence from controlled animal and human studies that sensory experiences early in life can influence flavor preferences and food acceptance [1–5]. But how these results relate to early sensory experiences and development of sensory preferences long-term in *human everyday settings* is still poorly understood. Previous research showed that breast-fed infants more rapidly accepted a new vegetable than formula-fed infants [6–8], and experience with a variety of vegetables at the start of complementary feeding increased intake of new foods a few weeks later [7, 9]. If these effects lasted for months and years then they might provide contexts for understanding how acceptance for a wide range of vegetables and perhaps other foods emerges and is maintained later on.

Breastfeeding is also associated with positive effects on later eating patterns [10–17]. Breastfeeding is linked to higher acceptance of new foods as they are introduced into the infant’s diet. In lactating mothers who consumed carrot juice for several days shortly after giving birth, the flavor was transferred to their milk and, at weaning, the infants showed “less negative facial expressions” when consuming cereal prepared with carrot juice than with unflavored cereal [18]. Besides this specific flavor-learning effect, breastmilk *per se* seems to favor acceptance of novel food perhaps because of the flavor variations in mother’s milk experienced by breast-fed infants [6]. Gerrish and Mennella [9] demonstrated that experiencing a high variety of vegetable purées (3 purées, changed each day over 9 days) at the start of complementary feeding increased intake of new foods over the next few days. These findings were later extended, showing that this increased acceptance of new vegetables and foods by infants who experienced a high variety of vegetable purées at the onset of complementary feeding lasted for at least 2 months [7]. Breastfeeding and early experience with flavor variety at the very beginning of complementary feeding interacted, in that infants who had been breast-fed and had then experienced a high variety of vegetables at weaning showed the most marked acceptance of new foods [7].

Several research groups have observed that, among infants of weaning age, repeated exposure to a new vegetable, even one that is initially disliked, can lead to increased acceptance of that vegetable [8, 19, 20]. For example, offering a new vegetable on 10 consecutive days to 4–6 month old infants led to a marked increase in intake between the first and the 10th day [8]. Parents asked to get their child to taste a previously disliked vegetable each day for 14 days reported a marked increase in liking for, and consumption of, the target vegetable [20]. Similarly, when a well-liked vegetable and an initially disliked vegetable were given to 7-month-old infants on alternate days over 16 days, by the 8th exposure to each, intake and liking of the two were similar [19]. Nine months later, most of the infants were still consuming and liking the previously disliked vegetable [19]. This persistence of increased acceptance was confirmed in children aged between 15 and 56 months, where repeated exposure to a new vegetable increased its intake 1 month and 6 months later. The effect of repeated exposure was more marked and more persistent in younger (<24 month-old) than in older (≥24 month-old) children [21]. Moreover, repeated exposure was sufficient to increase intake of a novel vegetable, regardless of the addition of a familiar liked taste or energy, in infants and in young children and this effect persisted for up to 6 months [22–26].

The present study examines the longer term effects of three contexts of exposure to food-related stimuli: breastfeeding, experiencing a variety of vegetables early in weaning and repeated exposure to an initially disliked vegetable, which, as shown above, have separate and interactive short term influences on subsequent acceptance of foods. It is a follow-up of the infants who participated in the two earlier studies described above [7, 19]. The objective was to evaluate the persistence of increased acceptance of vegetables at about 15 months, 3 and 6 years of age. At each follow-up age, a questionnaire was used to identify the number of vegetables offered and the number of vegetables eaten and liked by the child. At 6 years of age, consumption and liking tests in the laboratory were used to measure acceptance of new and familiar vegetables.

It is of obvious interest to establish if the increase in vegetable acceptance induced by any of these three contexts persists into childhood. The predictions we set out to test were that: (1) children who had been breast-fed and/or had experienced a high variety of vegetables at weaning should (a) accept more vegetables later on and (b) accept new vegetables more readily; (2) children who had experienced repeated exposure to an initially disliked vegetable and who had grown to like and accept it should continue to like and accept it at follow-up.

## Materials and Methods

### Participants

In the initial study there were 147 mother-infant dyads; 75 from Aalen in Germany and 72 from Dijon in France [7]. Infants’ age at the start of the study was 5.2±0.1 months (mean ± standard error, SE), consistent with previously reported ages for start of vegetable feeding in both regions. Of these, 107 (73%) participated in follow-up 1 (at age 14.6±0.2 months), 96 (65%) in follow-up 2 (at age 3.2±0.1 years) and 75 (51%) in follow-up study 3 (at age 6.0±0.04 years). Drop outs at these three time-points were fewer in Aalen (19%, 24%, and 41%) than in Dijon (36%, 47% and 57%). Reasons for drop out were: moved away, withdrew from the study, or did not return the questionnaire. The characteristics of mothers and infants in Dijon and Aalen in the two milk feeding groups and the three variety groups for the 4 study periods are shown in Tables 1 and 2.

**Table 1:** Characteristics of the Dijon sample split by mode of milk feeding and variety exposure group.

	Breast-fed infants ^4^			Formula-fed infants ^5^		
Variety group	No	Low	High	No	Low	High
**Mothers’ characteristics**						
Mother’s age (y)	29.3±1.13^1^	29.4±1.05	29.3±0.56	30.1±1.34	31.3±1.45	29.9±1.12
Mother’s BMI (kg/m^2^)	23.5±1.31	21.8±0.46	23.6±0.94	23.7±1.35	24.3±2.10	23.2±1.37
Primi/Multiparous (n)	10/8	6/6	7/8	3/5	3/6	5/5
**Variables related to the initial Study—Introduction of Vegetables**						
Number of infants (n)	18	12	15	8	9	10
Boys/Girls (n)	8/10	6/6	7/8	5/3	7/2	6/4
Infants’ age (mo)	5.2±0.22	5.1±0.33	5.5±0.24	5.7±0.47	5.0±0.19	4.9±0.20
Infants’ weight (kg)	6.7±0.21	6.9±0.24	7.0±0.22	7.3±0.50	6.8±0.17	6.9±0.24
Infants’ height (cm)	64.0±0.82	64.9±0.85	65.0±0.72	64.8±1.31	63.0±0.92	63.6±0.82
Breastfeeding duration (days)	126.3±11.58	111.8±14.76	108.5±12.67	1.8±1.75	3.9±2.06	3.5±1.88
**Variables related to children at the 1**^**st**^ **Follow-up**						
Number (n)	13	6	9	4	6	7
Age (mo)	14.9±0.43	13.9±0.55	15.1±0.41	15.1±0.54	14.8±0.42	14.2±0.33
z-BMI score ^2^	0.52±0.27	0.13±0.47	0.94±0.52	-0.33±0.49	0.80±0.41	0.34±0.22
Boys/Girls (n)	7/6	3/3	4/5	2/2	4/2	4/3
**Variables related to children at the 2**^**nd**^ **Follow-up**						
Number (n)	13	4	11	3	4	4
Age (mo)	45.3±1.94	37.8±1.17	49.0±2.46	48.7±3.57	43.9±2.83	46.7±3.09
z-BMI score ^2^	0.01±0.24	-0.24±0.62	0.46±0.25	1.16±0.63	0.19±0.37	0.27±0.58
Boys/Girls (n)	5/8	1/3	3/8	2/1	2/2	2/2
**Variables related to related to children at the 3rd Follow-up**						
Number (n)	12	6	4	3	2	4
Age (ys)	6.01±0.08	5.94±0.07	5.81±0.15	5.96±0.17	5.94±0.15	5.58±0.12
z-BMI score ^3^	0.18±0.30	-0.55±0.57	-0.31±0.70	0.25±1.10	1.26±1.60	-0.03±0.75
Boys/Girls (n)	5/7	4/2	3/1	2/1	1/1	2/2

^1^ Mean ± SE (all such values)

^2^ Calculated according to the WHO Child Growth Standards for boys and girls aged 0 to 60 months [27], from weight and height reported by parents from medical records

^3^ Calculated according to the WHO Child Growth Standards for 0–5 years [28], from weight and height reported by parents from medical records

^4^ Children breastfed for at least one month

^5^ Children breastfed for less than 15 days

**Table 2:** Characteristics of the Aalen sample split by mode of milk feeding and variety exposure group.

	Breast-fed infants ^5^			Formula-fed infants ^6^		
Variety group	No	Low	High	No	Low	High
**Mothers’ characteristics**						
Mother’s age (ys)	30.1±0.80^1^	34.6±1.28	32.5±1.35	29.9±1.50	29.8±1.23	31.8±1.22
Mother’s BMI (kg/m^2^)	23.7±1.08	23.7±0.94	26.0±1.48	24.8±1.18	28.7±2.12	25.3±1.18
Primi/Multiparous (n)	6/6	6/9	6/5	4/9	5/6	5/8
**Variables related to the initial Study—Introduction of Vegetables**						
Number of infants (n)	12	14	12	13	11	13
Boys/Girls (n)	6/6	7/8	5/6	7/6	6/5	7/6
Infants’ age (mo) ^2^	5.4±0.19	5.8±0.29	5.2±0.19	4.5±0.20	5.1±0.29	4.8±0.27
Infants’ weight (kg)	6.9±0.29	6.9±0.34	7.0±0.30	7.2±0.22	7.1±0.23	7.7±0.35
Infants’ height (cm)	64.4±0.79	65.0±0.94	65.0±0.94	65.1±0.80	64.8±0.90	64.2±0.78
Breastfeeding duration (days)	109.6±15.1	157.4±12.5	142.7±8.20	1.1±1.08	2.6±1.42	3.2±1.70
**Variables related to children at the 1**^**st**^ **Follow-up**						
Number (n)	12	11	10	9	9	10
Age (mo)	13.8±0.48	15.9±0.88	15.1±0.68	14.5±1.11	13.9±0.43	13.9±0.81
z-BMI score ^3^	-0.08±0.42	0.26±0.32	0.83±0.37	0.02±0.44	-0.56±0.31	0.16±0.39
Boys/Girls (n)	6/6	3/8	4/6	6/3	6/3	6/4
**Variables related to 2**^**nd**^ **Follow-up**						
Number (n)	10	8	12	9	9	9
Age (mo)	34.9±0.50	35.1±0.27	34.8±0.27	31.8±1.21	33.6±0.58	33.6±0.60
z-BMI score ^3^	-0.07±0.40	0.27±0.29	0.44±0.37	-0.06±0.30	-0.24±0.29	0.26±0.12
Boys/Girls (n)	5/5	3/5	6/6	5/4	6/3	6/3
**Variables related to 3**^**rd**^ **Follow-up**						
Number (n)	7	9	10	5	7	6
Age (ys)	6.04±0.15	6.34±0.08	6.19±0.10	5.94±0.22	6.16±0.12	5.93±0.07
z-BMI score ^4^	-0.37±0.41	0.50±0.41	-0.92±0.52	2.09±0.94	-0.41±0.70	2.09±0.94
Boys/Girls (n)	3/4	2/7	5/5	3/2	5/2	3/3

^1^ Mean ± SE (all such values)

^2^ There was a significant difference among infant’s age between the breast vs bottle-fed infants in the initial study [p = 0.002]

^3^ Calculated according to the WHO Child Growth Standards for boys and girls aged 0 to 60 months [27], from weight and height reported by parents from medical records

^4^ Calculated according to the WHO Child Growth Standards for 0–5 years [28], from weight and height reported by parents from medical records

^5^ Children breast-fed for at least one month

^6^ Children breastfed for less than 15 days

At each follow-up, the proportions of infants in each of the groups were similar to those in the initial study at 5 months [p>0.05, see Tables 1 and 2]. In addition, we repeated the original analysis of effects of breastfeeding and experience of different levels of vegetable variety, but included only the results for the sub-sample of children participating at each of the 3 follow-up studies. In each case, the same pattern of effects was observed, confirming that the children participating at each follow-up were representative of the original population.

### Ethics Statement

The study was approved in both regions by the local ethics committee (the ‘Comité de Protection des Personnes Est I Bourgogne’ in Dijon, France and Landesärztekammer in Aalen, Germany) and participating mothers signed an informed consent form.

### Experimental design

**Fig 1:**
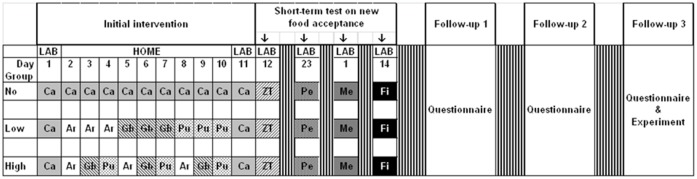
Experimental design. This includes the initial study with early exposure with flavor variety at the very beginning of complementary feeding and the short-term tests on new food acceptance. In the follow-up studies, we tested the persistence of these effects, measuring acceptance of vegetables when the children were about 15 months, 3 and 6 years of age. The measurements conducted at each follow-up are reported in the present paper. During the initial intervention (day 2 to 10) there were 3 groups of variety exposure at home: the ‘No’ variety group received carrot (Ca) every day, the 2 other groups were offered artichoke (Ar), Green beans (Gb) and pumpkin (Pu) for but the ‘Low’ variety group was given each purée for 3 consecutive days and there were daily changes for the ‘High’ variety group. In the initial study (7) acceptance of new foods (puréed zucchini-tomato mix (ZT) was evaluated at day 12, puréed peas (Pe) at day 23, meat purée (Me) when decided by the mother (so after a variable delay, mean ± SE: 21.7 ± 1.8 days) and fish purée (Fi) 13 days after this. In each of the 3 variety groups, there were ‘formula-fed’ and ‘breast-fed’ infants (i.e. who had been breast-fed for less than 15 days and more than 30 days, respectively).

The overall study design is shown in Fig 1. As described previously [7], the initial study was carried out at the very beginning of complementary feeding (when the children were ~5–6 months old) and the factors studied were type of milk feeding and experience with variety of vegetables early in weaning. The 2 levels of milk feeding were *breast-fed* and *formula-fed*. The 3 levels of variety experience were: *no variety*—carrot purée (Ca) given each day for 10 consecutive days; *low variety*—a first meal of carrot purée followed by artichoke purée (Ar), green beans (Gb), and then pumpkin (Pu) each given for 3 consecutive days, and *high variety*—carrot purée followed by the same 3 vegetables but with daily changes. In this initial study, we observed that in the short term (1) breastfeeding (as opposed to formula feeding) and (2) offering infants a high variety of vegetables at the onset of vegetable feeding increased acceptance of new foods, including vegetables offered during the following month [7]. In a subset of these infants we observed that offering an initially disliked and rejected vegetable purée at 8 subsequent meals markedly increased acceptance for that vegetable [19].

### Follow-up studies 1 and 2

Information about foods offered and food acceptance was collected using parent-completed questionnaires about nine months after the end of the initial study, i.e. when the children were on average 15 months (Follow-up 1) and 3 years (Follow-up 2). At follow-up 1, the children from the two countries were practically the same age; at follow-up 2 the children in Dijon were nearly a year older than the children in Aalen. Questionnaires (with detailed instructions and a reply envelope) were sent to the mothers to be completed at home. The questionnaires presented an exhaustive list of foods of different categories (beverages, fruit, cereals, starchy foods, protein-rich foods, mixed meals, dairy foods and cheeses, sweets), but only data concerning vegetables are reported here. For the French version of the questionnaire, the list of foods was based on earlier research conducted in France [29]. It was translated into German and adapted to German food patterns (and then back-translated to check there were no major discrepancies between the two versions). It was then tested with 20 mothers to ensure that the questions were well understood. Final versions were then prepared and administered. At 15 months, 36 different vegetables were listed for Aalen and 37 for Dijon (35 were common to Dijon and Aalen). At 3 years, the same 40 vegetables were listed for both regions. For each vegetable, the mother noted if, currently, the child (1) ate and liked it, (2) ate it but did not particularly like it, (3) had been offered it at least once but did not like it, (4) had been offered it but refused to taste it, or (5) had never been offered it. Acceptance was evaluated as the number of vegetables that the child “ate and liked”, and used as the dependent variable in the analyses of variance (ANOVAs) below.

### Follow-up 3

Follow-up 3 was carried out when the children were on average 6 years old. It involved two laboratory sessions each comprising 4 phases during which the children were offered different vegetables. Before the follow-up, each mother completed a questionnaire to assess the child’s current acceptance and consumption frequency during the past year of 40 different vegetables. In the first laboratory session, six different vegetables were chosen for each child as follows. One was a new vegetable for that child as identified from the questionnaire completed by the mother prior to the study session (examples of new vegetables were: fennel, broad beans and salsify in Dijon, parsley root, eggplant and artichoke in Aalen); four were liked familiar vegetables, also identified from the questionnaire (examples of liked familiar vegetables were: carrot, cauliflower, spinach, broccoli, green beans, leek, zucchini, bell pepper and pumpkin). The last one was the initially disliked vegetable for that child as identified in the initial study and which had been offered 8 times [19). This was included so that we could assess current liking specifically for the disliked vegetable. If there was no initially disliked vegetable, a vegetable classified in the questionnaire completed by the mother prior to the study session as either “offered at least once but not liked” or “offered it but refused to taste it” was chosen as a “filler” so that all children received 6 vegetables. In the second laboratory session, the six different vegetables were chosen for each child as follows: a second new vegetable for that child, four liked familiar vegetables (but different from those used in session 1), and the initially disliked vegetable or another “filler” chosen as described above for the first session.

#### Procedure

*Phase 1* served to familiarize the child with the study environment, the experimental room and the personnel carrying out the tests. It involved playing a ‘memory’ game with the experimenter in the presence of the mother to help the child adapt to the study environment. *Phase 2* allowed the child to practice using a 7-point hedonic scale [30], which ranged from ‘super bad’ (*‘trop trop mauvais’*; *‘super schlecht’*) = 1 to ‘super good’ (*‘trop trop bon’*; *‘super gut’*) = 7. In *Phase 3*, the children tasted, and rated liking for the 6 vegetables. During each of the two sessions (see above) the child was presented with a lunch tray with 6 small bowls (ramekins) each containing about 15 g of one of the 6 vegetables. The child was instructed to taste each vegetable and to rate, using the 7-point hedonic scale, how much s/he liked it. *Phase 4* consisted of a meal where the child was offered the same 6 vegetables and was free to eat as much of each as s/he wanted. Each vegetable (~50 g) was served on a small plate, the 6 plates being placed in a semi-circle in front of the child. In addition, 2 pieces of bread (~15 g), half a slice of cooked ham (~20 g) and a glass of water were provided as these are frequent lunch components in both regions. To complete the meal, children were offered a yogurt and a fruit purée. The child was given the following instructions: “For your meal, you can eat what you want, in the order you want, and you are not obliged to finish one plate you’ve begun. Don’t hesitate to take and move the plates.”

A given order of the different types of vegetables (i.e., familiar, new, disliked or “filler”) was chosen for each phase and applied to all children. Each session was videotaped by the experimenter. During the session, the mothers were in the room but 3 meters away from their child. She was instructed not to display any overt verbal or non-verbal response in direction of her child (other than mere occasional gazing at him/her). To distract her, she was asked to rate, on a 9-point hedonic scale, her child’s liking for each vegetable whilst the child was tasting it during Phase 3. During Phase 4, she was offered a light lunch composed of foods different from those served to the children.

#### Vegetable preparation and serving

All vegetables (100 g) were boiled in 500 ml of Evian water. After cooking, 2.5 g of butter and 0.6 g of salt were added per 100 g of cooked vegetables. The vegetables were placed in ramekins (phase 3) or on small plates (phase 4) heated to 40°C in a microwave oven, and then given to the child. In phase 4, each plate of vegetable was weighed before and after the session and the difference used to calculate the weight consumed.

### Effects of repeated exposure to an initially disliked vegetable on acceptance of that vegetable at follow-up

The participants in this part of the follow-up were a subgroup of the 147 mother-infant dyads in the original study conducted in two European regions [7]. On completion of that study, mothers who had identified at least one vegetable that their infant disliked so much that she had decided not to offer it again were asked to participate in a study where the infants were offered the initially disliked vegetable on 8 exposure days.

Intake and liking were measured at each meal. At the start of this stage of the study, there were 49 infants from Aalen and 40 from Dijon and their average age was 7.0 ± 0.9 months. After 7–8 repeated exposures, most (>80%) of the infants ate and liked the initially disliked vegetable as much as the liked vegetable [19]. In this follow-up study, we investigated if acceptance persisted into later childhood.

### Statistical analyses

#### Vegetable acceptance at follow-ups 1 and 2

To determine if there were significant effects of type of milk feeding (i.e. breast- *vs*. formula-feeding) and of early variety experience (i.e. high, low and no) on the mother-reported number of vegetables “eaten and liked” (questionnaire), ANOVAs were carried out. The model included breast vs. formula feeding, variety group, and region as factors, and interaction between type of milk feeding and variety. When non-significant, this interaction was removed in the final models. The number of vegetables offered was included as a covariate. This allows correction for potential differences between the different groups of children, in particular between the variety groups, as the initial allocation to a variety group may have affected longer term patterns of maternal feeding practices. It also allows correction for the slight difference in the number of vegetables listed in the French and in the German questionnaires at follow-up 1.

#### Vegetable acceptance at follow-up 3

Children’s vegetable acceptance during each laboratory session was assessed using liking rated by the child during phase 3, by intake (g) during phase 4, and by willingness to taste (i.e., the number of vegetables that were at least tasted). Correlation analyses showed there was a significant positive association between mother-reported and child-reported liking: r(73) = 0.86, p < 0.001, so only the latter ratings are reported here. The data for the two NEW vegetables were averaged as were data for the eight familiar vegetables and data for the two repeated measures for the initially disliked vegetable (DIS). For each dependent variable an ANOVA was performed. The model included 3 main effects (type of milk feeding, early variety experience, and region) and the interaction between type of milk feeding and variety. When non-significant this interaction was removed in the final models.

All data are presented as Least Square means (± SE) and the alpha value was set at 0.05. All statistics were carried out with the SAS statistics software (SAS Institute Inc., Cary, NC, USA).

## Results

### Participants

As noted above, not all the initial participants took part in the three follow-up studies. A series of χ^2^ tests confirmed that at each follow-up, the proportion of children remaining in each group was not significantly different from the proportion in the initial study (S1 Table). ANOVAs carried out on the intake and liking data from the initial study but using only results from infants participating in the follow-up studies yielded similar patterns of results to the initial analysis on the complete data set, showing that, at each follow-up, the children who participated were representative samples of those who took part in the initial study (S1 Fig).

### Follow-up 1

First, it is important to mention that there was no effect of type of milk feeding [p = 0.11], early variety experience [p = 0.51], and region [p = 0.48] on the number of vegetables offered to the children at follow-up 1, i.e. when the children were on average 15 months.

**Fig 2:**
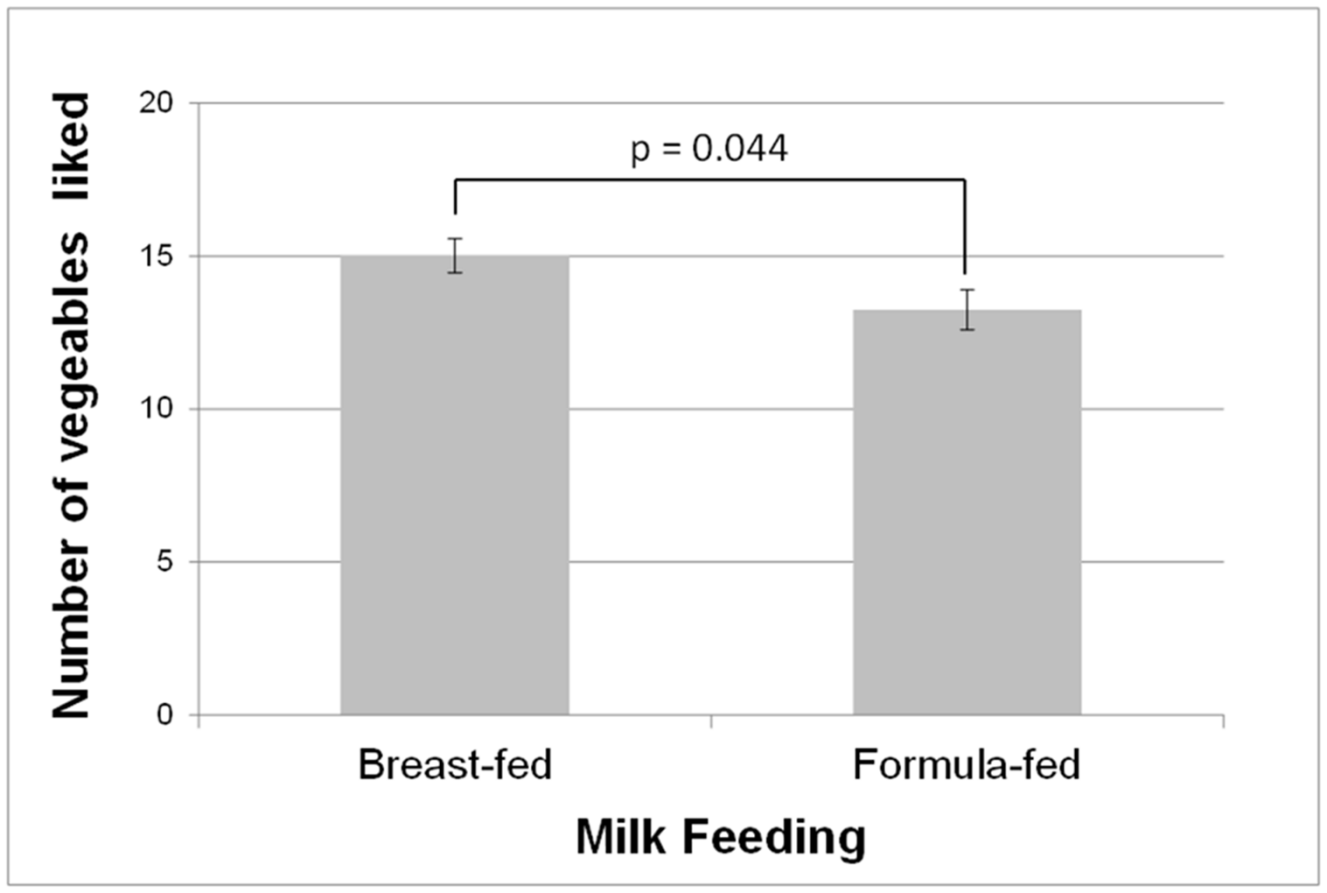
Higher number of vegetables liked by 15-month old children if they had been breast-fed. Mean ± SE of the maternal reports of number of vegetables eaten and liked at follow-up 1.

For the number of vegetables reported as eaten and liked by 15-month-olds there was a milk-feeding experience [F(1,100) = 4.16; p = 0.05] but not of early variety experience [p = 0.20]. As can be seen in Fig 2, children who had been breast-fed were reported to like more vegetables (15.0 ± 0.6 *vs*. 13.2 ± 0.7) than did those who had been formula-fed. There was also a significant effect of region [F(1,100) = 9.09; p = 0.004] with mothers in Dijon reporting that their children ate and liked more vegetables than mothers in Aalen reported for their children (15.4 ± 0.7 *vs*. 12.8 ± 0.6).

For continued acceptance of the initially disliked vegetable that, after repeated exposure, had become accepted and liked, the results were as follows: at 15 months, of the 80 children participating, 72 (85%) were still being offered the initially disliked vegetable. Of these, 57 (79%) were rated by the mother as eating and liking it, 8 (11%) as eating it but not particularly liking it and 7 (10%) as disliking or refusing it.

### Follow-up 2

At follow-up 2, the number of vegetables offered to the children did not differ by type of milk feeding [p = 0.28] or early variety experience [p = 0.43] but differed between regions [F(1,90) = 16.56; p = 0.0001] with more offered in Dijon (32.0 ± 0.9) than in Aalen (27.3 ± 0.7).

The number of vegetables “eaten and liked” differed significantly by region [F(1,90) = 10.72; p = 0.0015], but not by early variety experience [p = 0.0635], or type of milk feeding [p = 0.25]. Mothers in Aalen reported that their children ate and liked more vegetables than mothers in Dijon reported for their children (19.3 ± 0.9 *vs*. 14.3 ± 1.2).

Regarding continued acceptance of the initially disliked vegetable that, after repeated exposure, had become accepted and liked, at 3 years the results were as follows: of the 62 children still participating, 59 (82%) were still being offered the initially disliked vegetable. Of these, 43 (73%) were rated by the mother as eating and liking it, 11 (19%) as eating it but not particularly liking it and 5 (8%) as disliking or refusing it.

### Follow-up 3

At follow-up 3, i.e. when the children were on average 6 years old, the number of vegetables offered to the children (data extracted from mothers’ answers to the questionnaire) did not differ by type of milk feeding [p = 0.06] or early variety experience [p = 0.11] but differed between regions [F(1,70) = 70.78; p < 0.0001] with more offered in Dijon (33.9 ± 1.1) than in Aalen (22.2 ± 0.9).

**Fig 3:**
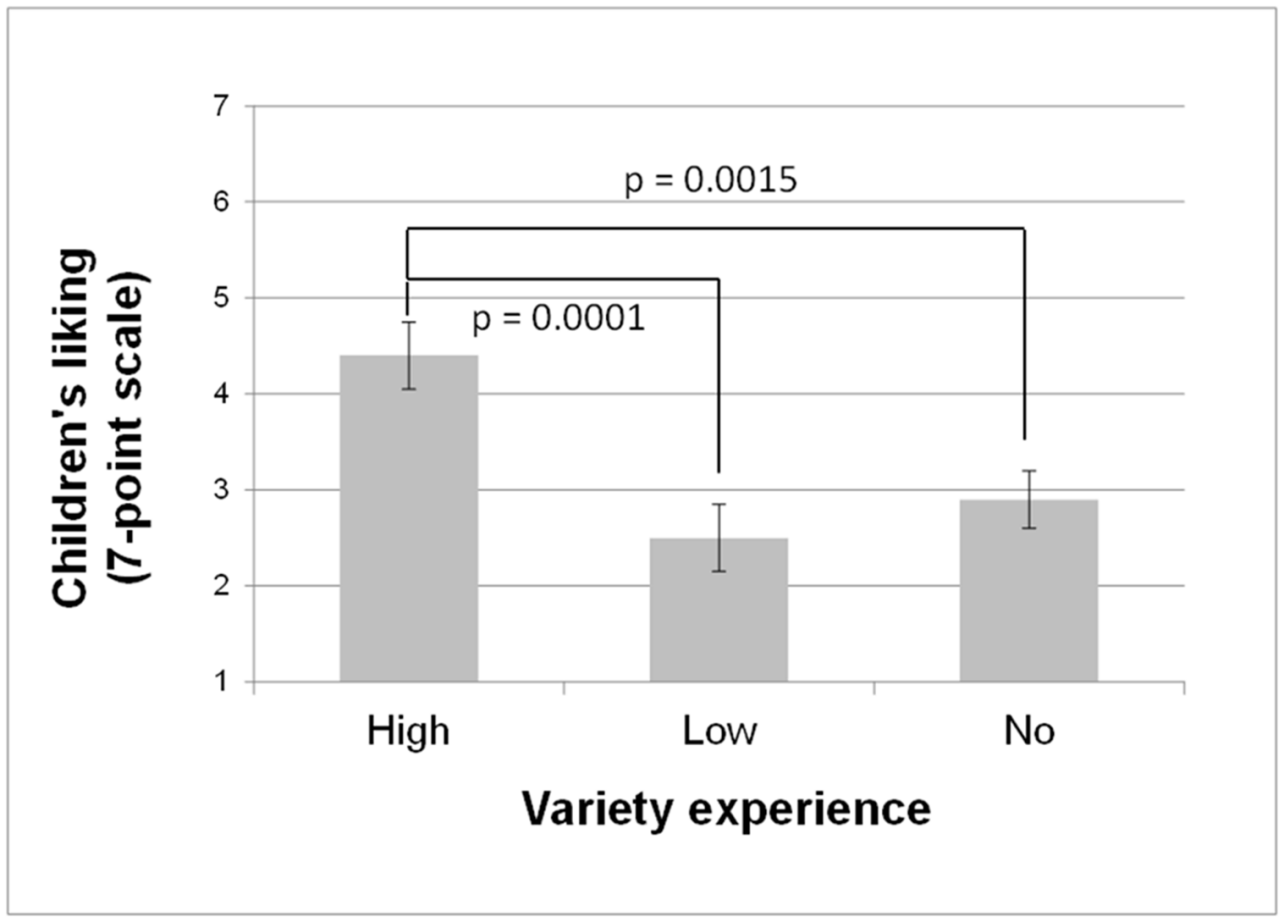
Early experience with high variety of vegetables is associated with higher liking of new vegetables. Liking (mean ± SE) at follow-up 3, i.e. when children were on average 6 years old, for the three experimental groups (no, low and high variety).

An ANOVA carried out on the mean child-reported liking for the new vegetables showed a significant early variety experience effect [F(2,70) = 9.41; p = 0.0002], but no effects of type of milk feeding or region [p = 0.92 and p = 0.20, respectively)]). As can be seen in Fig 3, children who had experienced a high variety of vegetables at weaning liked the new vegetables more than those who had low or no variety (scores of 4.4 ± 0.3 *vs*. 2.5 ± 0.3 and 2.9± 0.3).

**Fig 4:**
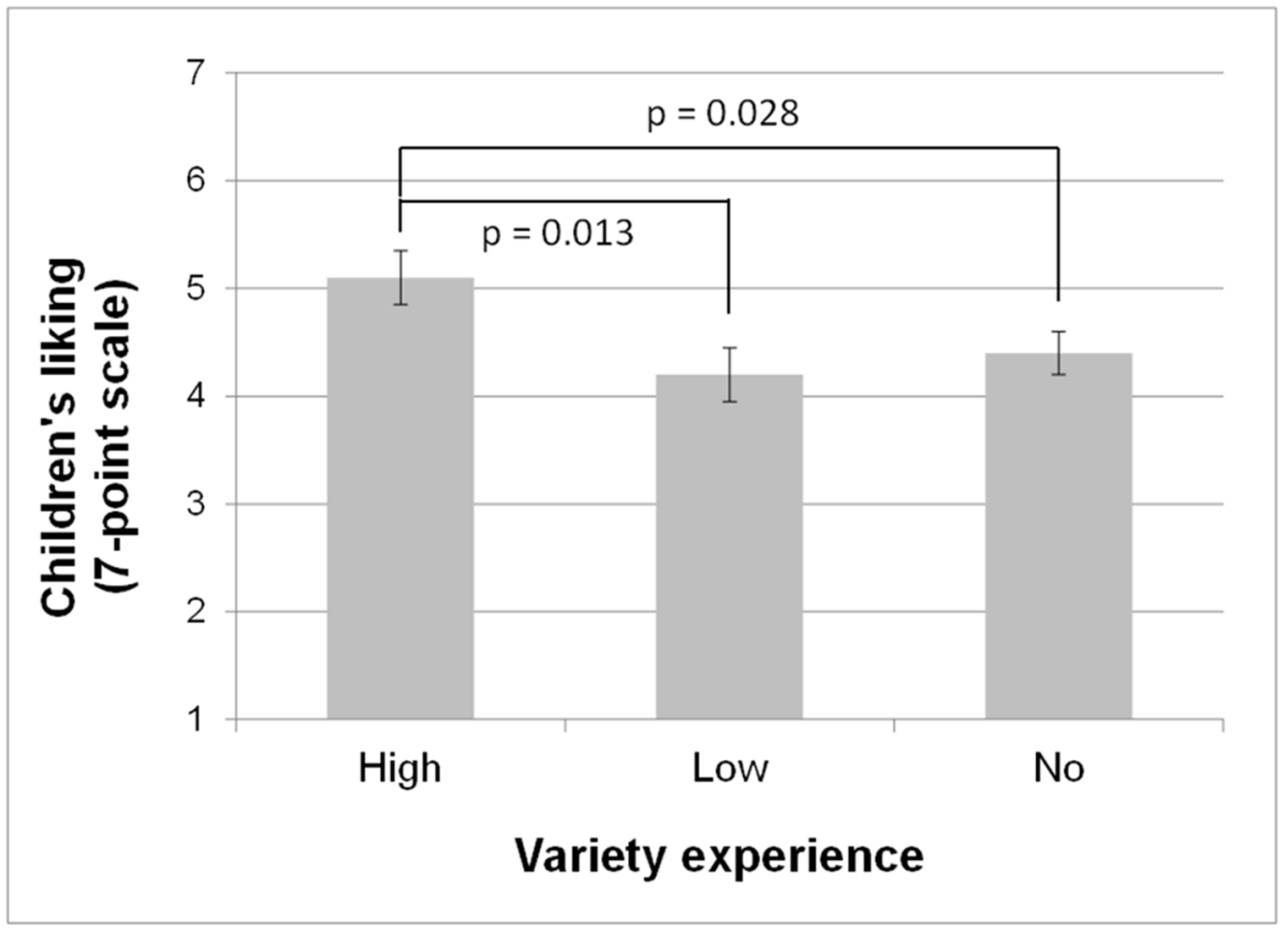
Early experience with high variety of vegetables is associated with higher liking of familiar vegetables. Liking (mean ± SE) at follow-up 3, i.e. when children were on average 6 years old, for the three experimental groups (no, low and high variety).

Mean liking for the familiar vegetables also showed a significant effect of early variety experience [F(2,70) = 3.90; p = 0.03]. As shown in Fig 4, children who had experienced a high variety of vegetables at weaning liked the new vegetables more than those who had low or no variety (scores of 5.1 ± 0.2 *vs*. 4.2 ± 0.2 and 4.4 ± 0.2). The effects of type of milk feeding or region were non-significant [p = 0.47 and p = 0.25, respectively].

**Fig 5:**
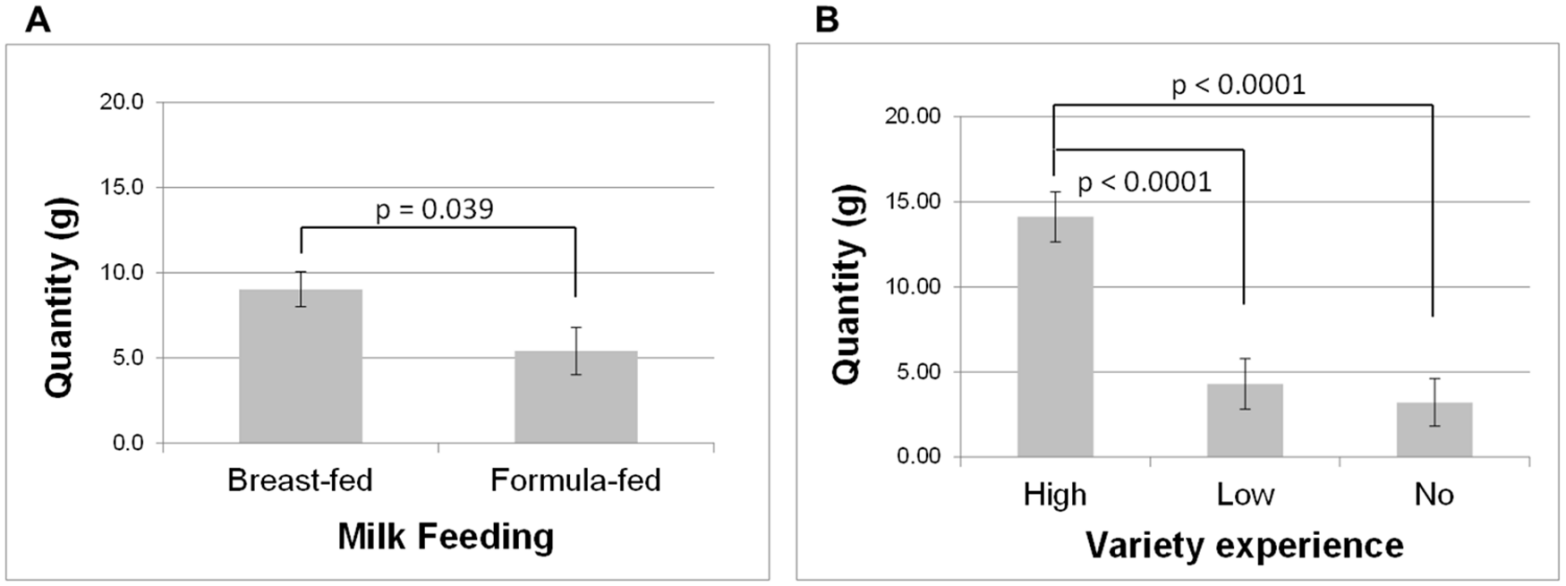
Early food-related variety experience is associated with higher intake of new vegetables. Intake (mean ± SE, in grams) at follow-up 3, i.e. when children were on average 6 years old. A: for breast-fed and formula-fed infants; B: for the three experimental groups (no, low and high variety).

An ANOVA carried out on the mean intake of the two new vegetables showed significant main effects of type of milk feeding [F(1,70) = 4.46; p = 0.04], of early variety experience [F(2,70) = 17.46; p < 0.0001] and of region [F(1,70) = 5.14; p = 0.03]. As can be seen in Fig 5, children who had been breast-fed consumed more of the new vegetables than did children who had been formula-fed (9.0 ± 1.0 g *vs*. 5.4 ± 1.4 g). Children who had experienced a high variety of vegetables at weaning ate more of the new vegetables than those who had experienced low or no variety in the initial study (14.1 ± 1.5 *vs*. 4.3± 1.5 and 3.2 ± 1.4 g, respectively). Lastly, children from Dijon consumed more of the new vegetables than did children from Aalen (9.1 ± 1.3 *vs*. 5.3 ± 1.1 g).

**Fig 6:**
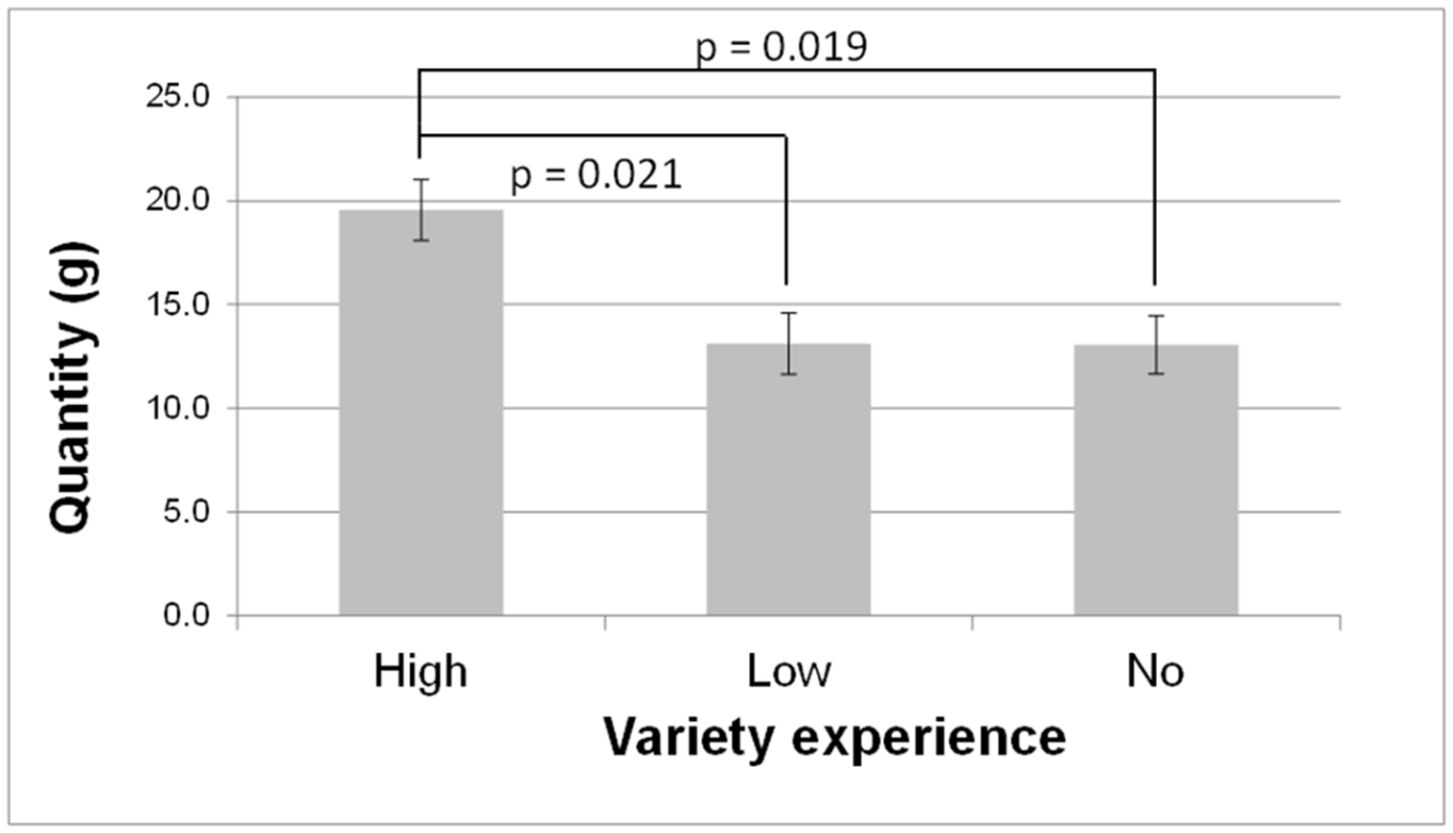
Early experience with high variety of vegetables is associated with higher intake of familiar vegetables. Intake (mean ± SE, in grams) at follow-up 3, i.e. when children were on average 6 years old, for the three experimental groups (no, low and high variety).

For mean consumption of the familiar vegetables there were significant main effects of early variety experience [F(2,70) = 3.78; p = 0.03] and region [F(1,70) = 16.94; p = 0.0001] but not of type of milk feeding. As can be seen in Fig 6, children who had experienced high variety of vegetables at the onset of weaning ate more of the familiar vegetables than those who had low or no variety (19.6 ± 2.0 *vs*. 13.1 ± 2.0 and 13.1 ± 1.9 g). Children from Dijon consumed more of the familiar vegetables than did the children from Aalen (19.9 ± 1.8 *vs*. 10.6 ± 1.4 g).

**Fig 7:**
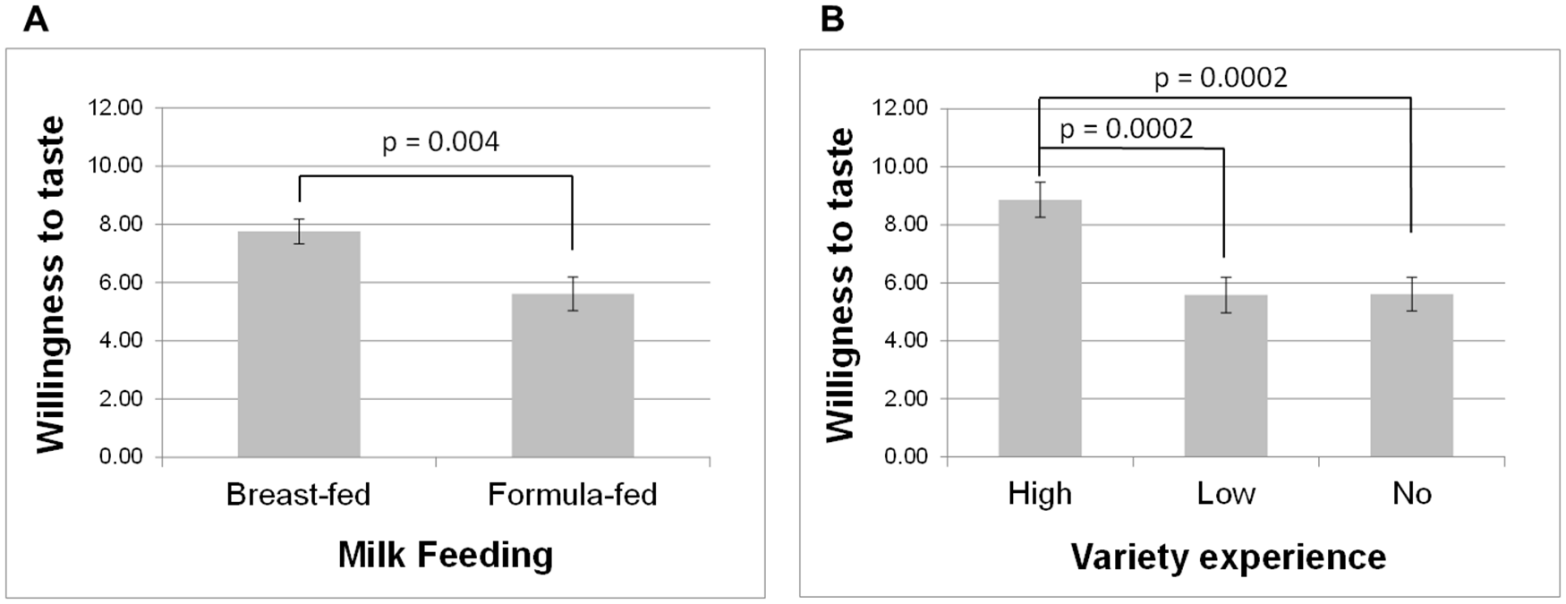
Early food-related variety experience is associated with higher willingness to taste vegetables. Mean (± SE) at follow-up 3, i.e. when children were on average 6 years old. A: for breast-fed and formula-fed infants; B: for the three experimental groups (no, low and high variety).

For willingness to taste (i.e., the number of vegetables that were at least tasted during the meal), there were significant main effects of type of milk-feeding [F(1,70) = 9.10; p = 0.004], early variety experience [F(2,70) = 10.07; p = 0.0001] and region [F(1,70) = 6.78; p = 0.02]. As can be seen in Fig 7, children who had been breast-fed tasted more vegetables than did formula-fed children (7.8 ± 0.4 *vs*. 5.6 ± 0.6), children who had experienced high variety of vegetables tasted more vegetables than those who had experienced low or no variety (8.9 ± 0.6 *vs*. 5.6 ± 0.6 and 5.6 ± 0.6, respectively) and children from Dijon tasted more vegetables than did children from Aalen (7.6 ± 0.6 *vs*. 5.8 ± 0.4).

For the initially disliked vegetable that, after repeated exposure, became accepted and liked, of the 53 children still participating at the 3rd follow-up, 49 (92%) were still being offered the initially disliked vegetable. Of these, 28 (57%) gave a positive liking score (≥5 on the 7-point scale), 4 (8%) gave it a neutral score (i.e., 4) and 17 (35%) disliked it (≤3).

## Discussion

This follow-up study confirms and extends our [7] and others’ (8,9) earlier observations on the effects of type of milk feeding and experience with different levels of vegetable variety at weaning on later acceptance of new vegetables. It provides evidence that both of these characteristics of early feeding practices have long lasting effects.

The long term effect of type of milk feeding on new vegetable acceptance was similar to that noted in the short term (i.e., one month after the beginning of complementary feeding). At 6 years, children who had been breast-fed consumed more of the new vegetables and were more willing to taste offered vegetables but there was no detectable effect on intake of familiar vegetables or on liking scores for new or for familiar vegetables. The pattern of these results is consistent with the argument that breast-fed infants are exposed to a diversity of flavors conveyed through their mother’s milk that are absent from formula milk and this early variety exposure acts to increase acceptance of novel flavors [6]. The results from questionnaires completed by the mothers concerning the number of familiar vegetables eaten and liked suggested a positive influence of breastfeeding on this measure at 15 months but not at 3 years.

The effect of variety offered at the start of complementary feeding was also similar to that observed in the short term. At 6 years, children who had been exposed to a high level of variety consumed more of, and had higher liking scores for new vegetables. They also showed greater acceptance for familiar vegetables and were more willing to taste vegetables (new or familiar) offered to them. In contrast, the questionnaire measures at 15 months and 3 years showed no significant effect on the number of familiar vegetables eaten and liked.

It is not possible to say whether these long term effects (of breastfeeding and of early variety exposure on new vegetable acceptance) were directly caused by the early experience of the infant or if subsequent behavior of the mother is also a factor. On one hand, there is evidence that flavor experiences during milk feeding do have direct long term effects on later flavor preferences [2, 3, 31, 32] while on the other hand, it is possible that a child’s greater enthusiasm for new vegetables at 6–7 months leads the mother to continue to offer new vegetables more often and with greater confidence, thus helping the child become “more familiar with novelty”.

Although this study showed that both breastfeeding (as opposed to formula feeding) and experiencing a high variety (as opposed to no or low variety) of vegetables at the start of complementary feeding led to measurable differences in acceptance of new vegetables at 6 years of age, this does not mean one can presume that these specific early experiences were the only influences on later acceptance. One possibility that we specifically examined was that the experience of offering her child more variety (or perhaps the infant’s short term increase in acceptance of new vegetables) might lead the mother in the high variety group to offer more vegetables over the long term. This did not appear to be the case because mothers in the high variety group did not report offering more vegetables to their offspring than did those in the low or no variety groups at any of the follow-up dates. This does not exclude the possibility that other, more subtle long term differences in maternal behavior occurred, but if they did, we were not able to identify them. Similarly, it is possible that mothers who breastfed, have different feeding practices that somehow led to increased acceptance of new foods. However, at none of the follow-up dates, did we observe mothers who had breastfed their infant reporting that they offered more vegetables to their offspring than mothers who had not breastfed. Nevertheless, again, this does not exclude the possibility that other non-controlled factors could have influenced children’s acceptance of vegetables.

As the results show, both variety experience at weaning and breast feeding were reliably associated with an increase in experimental measures of acceptance of new and of familiar vegetables at 6 years of age, but neither of them consistently predicted the ‘number of vegetables eaten and liked’ at 15 months and 3 years as measured by questionnaires. We used the mothers’ recall of the number of vegetables “eaten and liked” as a proxy measure for acceptance of new vegetables because the more easily the child accepts new vegetables, the more the mother is likely to offer them again, the more the vegetable will be liked (through repeated exposure) and the more the mother will be inclined to propose other new veg. In consequence, the child will have a larger repertoire of eaten and liked vegetables. The proxy measure was used because it was difficult to construct a viable question concerning how children easily accept new vegetables that was not open to serious bias. Moreover, mothers do not always correctly evaluate the children's degree of food preferences [33]. The absence of detectable influence of early variety exposure at follow-ups 1 and 2 and of breastfeeding at follow-up 2 could be linked to the tendency of 2–3 year-old children to refuse to eat not only novel foods but also foods they had previously consumed [34]. This period also corresponds to an increase in children’s non-compliance to parental requests, a developmental stage during which they tend to assert their autonomy [35]. Thus, these non-compliant behaviors could be more prominent at home than outside the home as in follow-up 3. Finally, the number of observations in each group was smaller than in the original study, so it is possible that reduced statistical power also contributed to the lack of significant differences at follow ups 1 and 2.

The observation that high variety experience also increased intake of, and liking for, more familiar vegetables needs to be explored further before drawing strong conclusions. It is indeed possible that, in the unfamiliar context of the procedure used (i.e., in the laboratory), the “familiar” vegetables may also have been perceived as somewhat new.

Concerning the number of vegetables offered, at follow-up 1, it was not significantly different according to the region, but it was higher in the Dijon group at follow-ups 2 and 3. This is in line with the practices observed during complementary feeding in the same regions in Germany and France: the number of vegetables and number of changes of vegetables were higher in Dijon than in Aalen [36]. Concerning children’s behavior, at follow-up 2 mothers in Aalen reported that their children ate and liked more vegetables than mothers in Dijon reported for their children; but at follow-up 3, differences between the two regions were not significant for liking whereas intake of new and familiar vegetables, and willingness to eat were higher in Dijon than in Aalen. This could reflect cultural differences in vegetable consumption during childhood: higher consumption of vegetables was reported in 11-year-old children in France compared to Germany with 51.8% and 40.2% girls, and 45.8% and 28.1% boys eating vegetables every day, respectively in France and Germany [37].

For the initially disliked vegetable that, after repeated exposure, became well accepted (see reference 19 for details), the follow-up results showed that most of the children continued to eat and like it. In practical terms, it was useful to demonstrate that repeated exposure to an initially disliked vegetable early in weaning, which increased its acceptance in the short term was also associated with continued acceptance in the long term (at 3 years, 73% of the mothers still reported that the initially disliked vegetable was well-liked and, at 6 years, 57% of the children, on tasting the vegetable, gave it a positive liking score). Since, however, there was no control group, further studies are needed before it will be possible to be sure how long the effect really lasts.

With respect to assessing if effects of repeated exposure to an initially disliked vegetable on acceptance at 7 months influence acceptance of the same vegetable at 6 years, it is important to remember that the way vegetables are presented changes with age. For example, 7-month-old infants are usually offered vegetable as purées but at 6 years the child is offered vegetables whole or cut into pieces, together with other foods and perhaps with added sauces and seasonings. This means that we cannot be sure that the version of the initially disliked vegetable offered at 15 months, 3 and 6 years is, for each child, equally representative of the version tasted at 7 months. It is, however worth noting that repeated exposure as a means of increasing acceptance of new foods seems to work over a range of ages. Several studies have reported the phenomenon in weanlings [8, 26]. Ahern et al. [21] observed a persistent (for 6 months) increase in acceptance of a novel root vegetable (celeriac, swede and turnip) after 6–8 exposures in 15–56 month-old children; a persistent effect (for 6 months) was also observed in pre-school children [22, 24, 25], while Wardle et al. [20] found that repeated exposure increased intake of, and liking for, a novel vegetable (red pepper) in 5–7 year olds. The importance of this effect has considerable practical implications. As noted earlier with a large US sample [38], a majority of parents reported that they offered a refused food less than 5 times before they decided the child disliked it. In the same regions in Germany and France where the present study was conducted, we observed that, if infants initially disliked a vegetable, most mothers (85%) offered it at no more than 3 subsequent meals before giving up and deciding not to offer it again [36]. The results of the present study suggest that, rather than giving up after 2–3 tries, it is well worth offering an initially disliked vegetable up to 8–10 times, without pressuring the child to eat it, because it is likely to be followed by adequate acceptance well into childhood.

One problem with long-term follow-up studies is that there is inevitably some attrition among the participants. Here, the losses were considerable: 27% at 15 months; 35% at 3 years and 49% at 6 years. The proportion of losses, however, was similar in the different cells of the experimental design. An analysis of new vegetable intake and liking carried out on the initial data collected when the infants were weanlings, but using only those infants who later on participated in the follow-up studies, confirmed that the participants at each stage of follow-up were representative of the initial population in that their initial patterns of results were similar.

As increased early variety increases acceptance of new foods, this may increase the size of the food repertoire and consequently the variety of the diet. This could have a positive effect as it could contribute to increase the nutritional variety of the diet.

## Conclusions

This study shows the effectiveness of breastfeeding and early experiences with vegetable variety during complementary feeding in promoting acceptance of new vegetables into childhood. In addition, it demonstrates that offering an initially disliked vegetable to infants at 8 subsequent meals reliably increases consumption of, and liking for, that same vegetable for up to 6 years. That these three effects are long-lasting and robust provides the foundation for evidence-based recommendations to help parents increase healthy eating habits in their children.

## Supporting Information

S1 Fig Results of the acceptance (intake, liking score given by the mother, mean ± SE) of the 2 new vegetables which were offered within one month after the beginning of the initial intervention for the initial sample and for the samples of infants participating at each follow-up. For intake, means are presented by type of variety experience x type of milk feeding as the interactions between these factors was significant or tended to be significant [p = 0.008 for the initial sample, p = 0.06 at follow-up 1, p = 0.05 at follow-up 2 and p = 0.09 at follow-up 3]. For liking, means are presented for each level of type of variety experience and each level of type of milk feeding as the interaction between these factors was never significant and thus was removed from the model.
(PDF)

S1 Table Chi-2 values for the comparisons of the Dijon and Aalen samples of children participating at each follow-up with the initial sample of children in terms of frequency for each type of milk feeding and in terms of each type of variety experience. (PDF)
