# Supplementary material for: The Lasting Influences of Early Food-Related Variety Experience: A Longitudinal Study of Vegetable Acceptance from 5 Months to 6 Years in Two Populations
Source: PLoS One. 2016 Mar 11;11(3):e0151356. doi: 10.1371/journal.pone.0151356 (PMC4788196; doi:10.1371/journal.pone.0151356)

S1 Fig. Results of the acceptance (intake, liking score given by the mother, mean ± SE) of the 2 new vegetables which were offered within one month after the beginning of the initial intervention for the initial sample and for the samples of infants participating at each follow-up. For intake, means are presented by type of variety experience x type of milk feeding as the interactions between these factors was significant or tended to be significant (p=0.008 for the initial sample, p=0.06 at follow-up 1, p = 0.05 at follow-up 2 and p= 0.09 at follow-up 3). For liking, means are presented for each level of type of variety experience and each level of type of milk feeding as the interaction between these factors was never significant and thus was removed from the model.

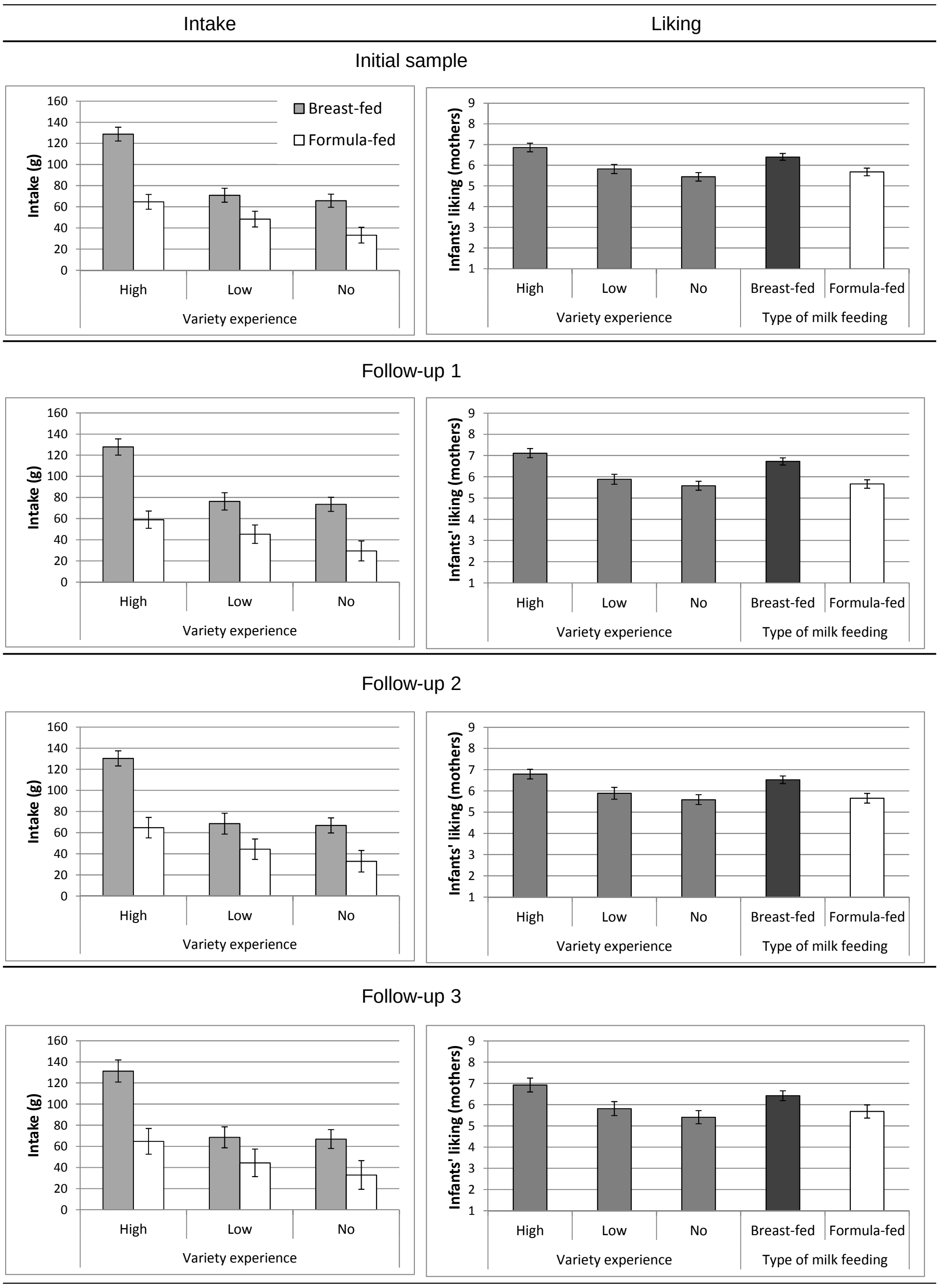

Supplement: S1 Fig — For intake, means are presented by type of variety experience x type of milk feeding as the interactions between these factors was significant or tended to be significant [p = 0.008 for the initial sample, p = 0.06 at follow-up 1, p = 0.05 at follow-up 2 and p = 0.09 at follow-up 3]. For liking, means are presented for each level of type of variety experience and each level of type of milk feeding as the interaction between these factors was never significant and thus was removed from the model. (PDF) [file pone.0151356.s001.pdf]
