# Supplementary material for: The Lasting Influences of Early Food-Related Variety Experience: A Longitudinal Study of Vegetable Acceptance from 5 Months to 6 Years in Two Populations
Source: PLoS One. 2016 Mar 11;11(3):e0151356. doi: 10.1371/journal.pone.0151356 (PMC4788196; doi:10.1371/journal.pone.0151356)
Supplement: S1 Table — (PDF) [file pone.0151356.s002.pdf]

S1 Table. Chi-2 values for the comparisons of the Dijon and Aalen samples of children participating at each follow-up with the initial sample of children in terms of frequency for each type of milk feeding and in terms of each type of variety experience.

Region	Frequency compared	Follow-up 1	Follow-up 2	Follow-up 3
Dijon	Breast- vs. formula-feed	0.00	0.97	0.68
	No vs low vs high variety	0.09	0.98	1.44
Aalen	Breast- vs. formula-feed	0.16	0.05	0.79
	No vs low vs high variety	0.02	0.24	0.48

All values were NS
